# Supplementary material for: DispHScan: A Multi-Sequence Web Tool for Predicting Protein Disorder as a Function of pH
Source: Biomolecules. 2021 Oct 28;11(11):1596. doi: 10.3390/biom11111596 (PMC8616002; doi:10.3390/biom11111596)
Supplement: Supplementary file 1 [file biomolecules-11-01596-s001.zip › biomolecules-1431198-supplementary.pdf]

Supplementary material for

DispHScan: a multi-sequence web tool for predicting protein disorder as a function of pH

Carlos Pintado-Grima, Valentín Iglesias, Jaime Santos, Vladimir N. Uversky and Salvador Ventura*

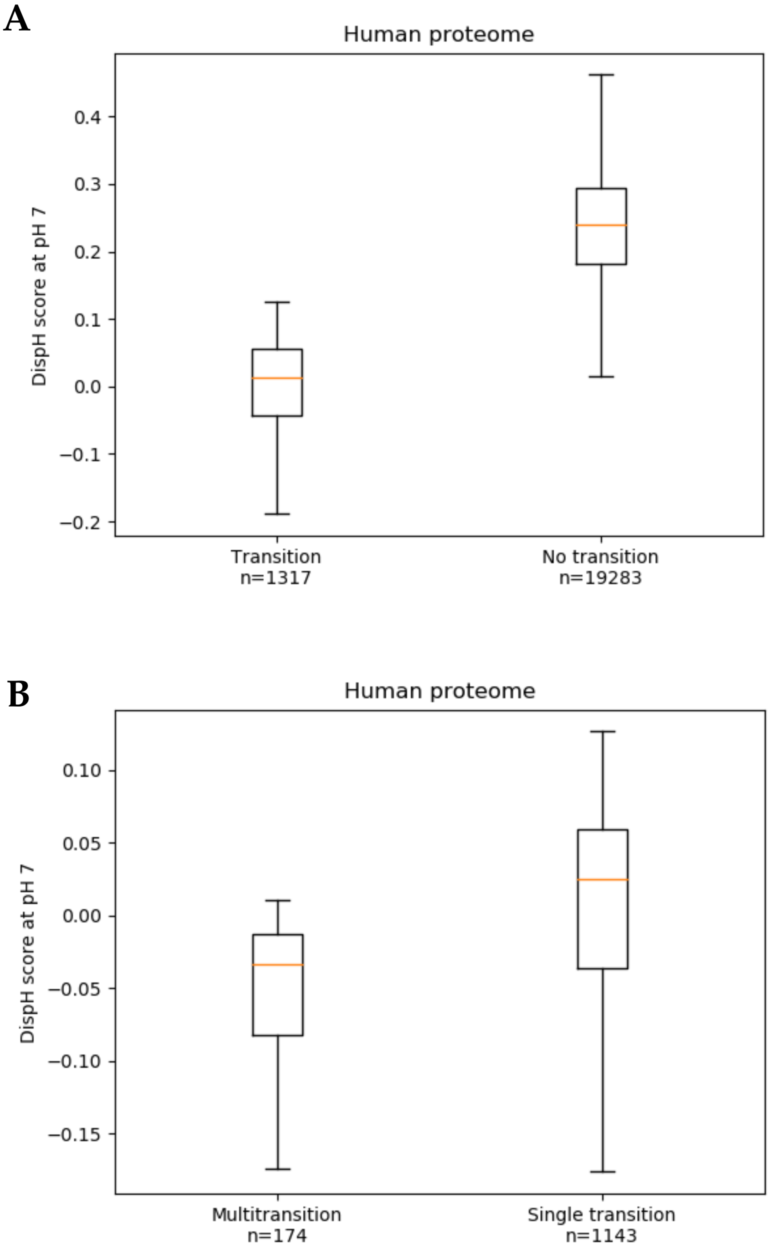

Figure S1. Median DispH scores obtained for the human proteome (UP000005640_9606) analyzed with DispHScan at pH 7 for (A) transitioning (0.014) and non-transitioning proteins (0.240) and (B) multi-transitioning (-0.034) and single-transitioning proteins (0.024).
